# The Ratio of NT-proBNP to CysC^1.53^ Predicts Heart Failure in Patients With Chronic Kidney Disease

**DOI:** 10.3389/fcvm.2021.731864

**Published:** 2021-11-12

**Authors:** Sheng Wang, Ming Li, Xiangyu Wang, Jing Luo, Yulin Zou, Yang Hu, Xingtai Liu, Hua Ao, Xueer Yao, Chufeng Li, Tingting Yang

**Affiliations:** ^1^Department of Clinical Laboratory, The Third Clinical Medical College of the Three Gorges University, Gezhouba Central Hospital of Sinopharm, Yichang, China; ^2^Department of Nephrology, The Third Clinical Medical College of the Three Gorges University, Gezhouba Central Hospital of Sinopharm, Yichang, China; ^3^Department of Neurology, The Third Clinical Medical College of the Three Gorges University, Gezhouba Central Hospital of Sinopharm, Yichang, China; ^4^Department of Medical Business, The Third Clinical Medical College of the Three Gorges University, Gezhouba Central Hospital of Sinopharm, Yichang, China; ^5^Department of Pharmacy, The Third Clinical Medical College of the Three Gorges University, Gezhouba Central Hospital of Sinopharm, Yichang, China; ^6^Department of Pain and Rehabilitation, The Third Clinical Medical College of the Three Gorges University, Gezhouba Central Hospital of Sinopharm, Yichang, China; ^7^Department of Ultrasound, The Third Clinical Medical College of the Three Gorges University, Gezhouba Central Hospital of Sinopharm, Yichang, China; ^8^Department of Endocrinology, The Third Clinical Medical College of the Three Gorges University, Gezhouba Central Hospital of Sinopharm, Yichang, China

**Keywords:** chronic kidney disease, heart failure, NT-proBNP, CysC, combined diagnostic index

## Abstract

**Background:** The N-terminal pro B type natriuretic peptide (NT-proBNP) is important for prognosis of heart failure in patients with chronic kidney disease (CKD). However, the NT-proBNP level is easily affected by renal insufficiency, which limits its clinical use.

**Methods:** This study included 396 patients with CKD. Plasma levels of NT-proBNP and cystatin C (CysC) were measured during hospitalization. The echocardiographic parameters were also detected. Patients were divided into the heart failure group and control group according to the European Society of Cardiology Guideline on Chronic Heart Failure 2021. Multiple modeling analysis of the values of NT-proBNP and CysC, including NT-proBNP/Cysc^n^ and NT-proBNP/n^CysC^ was performed. The receiver operating characteristic (ROC) curve, combined with the cardiac function, was used to determine the formula with the best diagnostic efficiency. Then, the sensitivity and specificity of new predictors for cardiac insufficiency in CKD patients were calculated. Pearson correlation analysis was used to analyze the relationship between new predictors and the NT-proBNP level. The clinical data of CKD patients from another local hospital were used to validate the new predictors and the cut-off values.

**Results:** An elevated NT-proBNP/CysC^1.53^ ratio was an independent risk factor for cardiac dysfunction in CKD and the best predictor derived from multiple modeling analysis. There was no correlation between the NT-proBNP/CysC^1.53^ ratio and the NT-proBNP level (*r* = 0.376, *p* = 6.909). The area under the ROC curve for the NT-proBNP/CysC^1.53^ ratio was 0.815 (95% confidence interval: 0.772–0.858), and for a cut-off point of 847.964, this ratio had a sensitivity of 78.24%, and a specificity of 69.44%. When applied to the data of CKD patients from another local hospital, the NT-proBNP to CysC^1.53^ ratio had a sensitivity of 70.27% and a specificity of 67.74%.

**Conclusion:** The NT-proBNP to CysC^1.53^ ratio was superior to NT-proBNP alone for predicting cardiac dysfunction in patients with CKD.

## Introduction

Chronic kidney disease (CKD) is diagnosed when the estimated glomerular filtration rate (eGFR) is <60 mL/min/1.73 m^2^ for 3 consecutive months, or abnormal renal structure or function other than decreased eGFR for over 3 months (1). The main cause of death among patients with CKD is cardiovascular disease, including myocardial infarction and heart failure (HF) (2–4). When HF occurs in CKD patients, the retention of sodium and fluid leads to increases in vascular tension and cardiac preload (5). The increase in ventricular pressure induces the release of biomarkers, such as natriuretic peptide (6). Therefore, biomarkers of myocardial stretching are often used for the diagnosis and prognosis of HF (7). Brain natriuretic peptide (BNP) and N-terminal pro B type natriuretic peptide (NT-proBNP) are important indicators for the diagnosis, prediction, and treatment evaluation of HF, and NT-proBNP is superior to BNP in this prognostic assessment (8–11). Although they both are important indicators of HF (12), NT-proBNP is rarely used as a diagnostic biomarker for HF in patients with end-stage renal disease (ESRD) (13), because ESRD patients without HF have high levels of NT-proBNP due to decreased renal elimination, volume overload, hypertension, and increased left ventricular hypertrophy (14). NT-proBNP is mostly eliminated by glomerular filtration, which explains the strong influence of renal function on NT-proBNP levels (15).

When using NT-proBNP for the diagnosis of HF in patients with renal insufficiency, the diagnostic cut-off value must be adjusted according to the eGFR (16). The cut-off value of NT-proBNP for the diagnosis of acute HF (AHF) in patients with CKD stages 3–5 (eGFR <60 mL/min/1.73m^2^) is higher than that in patients with CKD stages 1–2 (eGFR >60 mL/min/1.73m^2^), ranging from 1,200–6,000 pg/mL, and both points are higher than the standard “Januzzi cut-off point.” In general, the specificity and sensitivity of the NT-proBNP concentration for the diagnosis of AHF in patients with CKD stages 3–5 are low (7). To date, there have been no large-scale prospective clinical trials determining the diagnostic cut-off value of NT-proBNP for HF in CKD patients (17).

It is important to use a reliable serological indicator to predict HF in CKD patients with different eGFRs. Low molecular-weight proteins have been used to estimate the value of eGFR (18), and the most promising biomarker is cystatin C (CysC) (19). CysC is a non-glycosylated, 13-kDa basic protein that belongs to the cystatin superfamily of cysteine protease inhibitors. It is produced by all nucleated cells, unaffected by muscle mass (unlike creatinine) (20), and considered a replacement for serum creatinine (sCr) for the estimation of eGFR (21). CycC is also used for early diagnosis of renal damage (22).

In the present study, we investigated the prognostic potential of the ratio of NT-proBNP to CysC compared with NT-proBNP alone for cardiac dysfunction in Chinese CKD patients.

## Materials and Methods

### Study Population

The clinical records of 938 adults who underwent serum CysC measurement and cardiac biomarker measurement simultaneously between September 2020 and August 2021 in Gezhouba Central Hospital of Sinopharm, China, were retrospectively reviewed. Individuals who were diagnosed with CKD, aged above 18 years, and had complete medical records were eligible for this study. This study was approved by the Hospital Ethics Committee, and informed consent was obtained from all patients. To minimize the confounding effects of circulating biomarkers, patients who met any of the following criteria were excluded: (1) diagnosed with acute myocardial infarction, acute HF, atrial fibrillation, or unstable angina within 1 month of enrollment; (2) diagnosed with malignant arrhythmia or hemodynamic changes; (3) diagnosed with primary liver disease complicated with HF; (4) diagnosed with an end-stage malignant tumor; (5) diagnosed with primary thyroid disease; (6) pregnant; or (7) diagnosed with blood system diseases. Finally, 396 patients were included in the study. Among them, there were 123 (31.06%) cases of diabetic nephropathy, 75 (18.94%) cases of chronic glomerulonephritis, 87 (21.97%) cases of hypertensive nephropathy, 41 (10.35%) cases of nephrotic syndrome, 39 (9.85%) cases of IgA nephropathy, 12 (3.03%) cases of polycystic kidney, 8 (2.02%) cases of after renal transplantation, and 11 (2.78%) cases of other types of kidney diseases. A total of 98 (24.74%) patients had been diagnosed with HF during previous hospitalization.

During hospitalization, all patients with CKD were given a low-salt (3–5 g/day), low-fat, high-quality, low-protein diet and medications to control blood pressure, blood glucose, and blood lipids. All patients were given 3–5 compound ketoic acid tablets orally (three times per day). Patients with proteinuria (*n* = 156) were given huangkui capsule (three times per day). Seventy-two patients received hormone and immunosuppressant therapy. Spironolactone (20 mg, once daily) was routinely given to CKD patients with previously diagnosed HF. Beta-blockers (12.5–25 mg, twice daily) and angiotensin-converting enzyme inhibitor (ACEI)/angiotensin receptor blocker (ARB) drugs were used in antihypertensive treatment for patients with eGFR >30%. Calcium channel blocker drugs were used in antihypertensive treatment for patients with eGFR <30%. All enrolled patients were treated with cardiac intensification and diuretic therapy for acute HF during the observation period. Serum potassium was regularly monitored in all patients treated with concomitant spironolactone and angiotensin converting enzyme inhibitors or angiotensin receptor blocker.

During the follow-up period, 57 patients showed clinical symptoms of HF, including 25 patients with newly diagnosed HF and 32 patients diagnosed with HF during previous hospitalization. Thirty-nine patients required hospitalization, while the other 18 were in stable condition after outpatient cardiotonic and diuretic treatment. During the follow-up period, all patients with HF clinical symptoms were continually followed after their condition became stable, and HF was treated according to aforementioned regimes. Serum potassium was routinely monitored in all patients treated with concomitant spironolactone and ACEI/ARB.

### Biomarker Assessment

The levels of highly sensitive cardiac troponin T (hs-cTnT) and NT-proBNP were measured by the Cobas 601 automatic chemiluminescence analyzer (Roche, Inc.). The levels of CysC, serum urea and sCr were measured using the 7600 automatic biochemical analyzer (Hitachi, Inc.). The levels of hemoglobin (Hb) were measured using the XN-1000 automatic blood cell analyzer (Sysmex, Inc.).

### Echocardiography

On the day that blood tests were performed, echocardiography also was performed using the GE Vivid E90 Ultrasound System (M5S probe, 1.7~3.3 MHz; GE Healthcare) with participants laying on their left side and staying calm during the test. The following parameters were measured: left ventricular end-diastolic dimension (LVDd), left ventricular end-systolic dimension (LVDs), left ventricular ejection fraction (LVEF), left atrial volume index (LAVI), left ventricular mass index (LMVI), global area strain (GAS), global longitudinal strain (GLS), left ventricular fractional shortening (FS), stroke volume (SV), and the echocardiographic ratio of early diastolic mitral inflow velocity to early diastolic mitral annulus velocity (E/e').

### Diagnostic Criteria for HF

HF was diagnosed based on the symptoms and/or signs of HF, echocardiography report, and the Heart Failure Association (HFA)-PEFF (P, pre-test assessment; E, echocardiography and natriuretic peptide score; F1, functional testing; F2, final etiology) diagnostic algorithm (23). The algorithm consists of three domains: functional, morphological, and biomarker domains. For each domain, 2 points are scored when the main criteria are met, while 1 point is scored when minor criteria are met. Points from different domains are then summed. A total score of ≥5 is considered a diagnosis of HF preserved ejection fraction (HFpEF); while a score of ≤1 indicates an unlikely diagnosis of HFpEF (24). The diagnosis of HF with mildly reduced ejection fraction (HFmrEF) requires the presence of symptoms and/or signs of HF, the LVEF between 40 and 50%, elevated levels of natriuretic peptides (BNP ≥35 pg/mL or NT-proBNP ≥125 pg/mL), and other evidence of structural heart disease (25). The diagnosis of HF with reduced ejection fraction (HFrEF) requires the presence of symptoms and/or signs of HF and a reduced ejection fraction (LVEF ≤40%) (25).

### Statistical Analysis

SPSS 22.0 software (SPSS, Inc.) was used for data analysis. The levels of NT-proBNP were log-transformed, and then statistical analysis was performed to eliminate the influence of extreme values. Normally distributed data are expressed as $\bar{x}$ ± *s*. Data that were not normally distributed [i.e., NT-proBNP levels, CysC levels, sCr, eGFR, and the ratio of NT-proBNP/CysC^1.53^ (new predictors)] are expressed as median (P25~P75) values. An independent sample *t*-test was used to compare the results between two groups. Multiple modeling analysis of the values of NT-proBNP and CysC, including NT-proBNP/CysC^n^ and NT-proBNP/n^CysC^, was performed. The ROC curve, combined with the cardiac function, was used to determine formula with the best diagnostic efficiency. Then, the sensitivity and specificity of new predictors for cardiac insufficiency in CKD patients were calculated. ROC curve analysis was used to analyze echocardiography indicators with statistical significance. The area under the ROC curve (AUC) was calculated to evaluate the ability of these factors to predict HF in patients with CKD. The optimal diagnostic cut-off point was determined by the “Youden index” (sensitivity + specificity−1). The significance level was α = 0.05, and *p* < 0.05 was considered statistically significant.

## Results

The baseline characteristics of 396 hospitalized patients with CKD are summarized in Table 1. Patients were divided into two groups according to their cardiac function assessed following the European Society of Cardiology (ESC) Guidelines for Chronic Heart Failure 2021: the HF group (*n* = 216) and the control group (*n* = 180).

**Table 1:** Clinical characteristics of CKD patients at baseline.

**Variables**	**HF group** **(*n* = 216)**	**Control group** **(*n* = 180)**	***t-value***	***p*-value**
Age (years)	74.296 ± 8.938	72.872 ± 9.777	1.513	0.291
Male [cases (%)]	132 (61.11)	115 (63.89)	0.323	0.322
BMI (kg/m^2^)	25.195 ± 3.088	24.442 ± 2.742	2.540	0.186
LVDd (mm)	51.621 ± 9.033	50.204 ± 8.028	1.634	0.497
LVDs (mm)	34.726 ± 10.030	32.334 ± 8.871	2.488	0.131
LVEF (%)	56.661 ± 11.766	62.446 ± 8.347	5.537	0.000
SV	73.819 ± 24.478	75.2905 ± 24.151	0.599	0.489
FS	32.257 ± 8.313	35.233 ± 7.985	3.311	0.279
E/e'	18.728 ± 4.690	14.902 ± 2.060	10.157	0.000
GAS (%)	−25.911 ± 3.118	−26.672 ± 2.731	2.560	0.016
GLS (%)	−14.958 ± 2.589	−15.258 ± 2.337	1.200	0.019
LAVI (mL/m^2^)	38.248 ± 3.757	33.175 ± 3.766	12.310	0.003
LVMI (g/m)	110.558 ± 11.946	108.465 ± 13.760	1.600	0.019
Hb (g/L)	102.944 ± 24.874	105.633 ± 25.148	1.066	0.549
serum urea (mmol/L)	14.602 ± 6.787	14.100 ± 5.281	0.808	0.013
sCr (μmol/L)	257.00 (176.25, 409.00)	202.25 (279.00, 390.00)	0.507	0.010
CysC (mg/L)	3.160 (2.183, 4.703)	3.295 (2.183, 4.290)	0.651	0.057
eGFR (mL/min/1.73 m^2^)	15.442 (9.341, 25.444)	15.534 (10.595, 26.007)	0.164	0.814
hs-cTnT (μg/L)	0.018 ± 0.007	0.015 ± 0.007	4.087	0.001
NT-proBNP (pg/mL)	8,505 (4553, 16128)	4,271 (2203, 6878)	6.728	0.000
NT-proBNP/CysC^1.53^	1282.764 (843.440, 2117.184)	636.921 (307.293, 1037.159)	8.065	0.000

The ROC curve showed that the cut-off value of the NT-proBNP/CysC^1.53^ ratio was 847.964.

**Figure 1:**
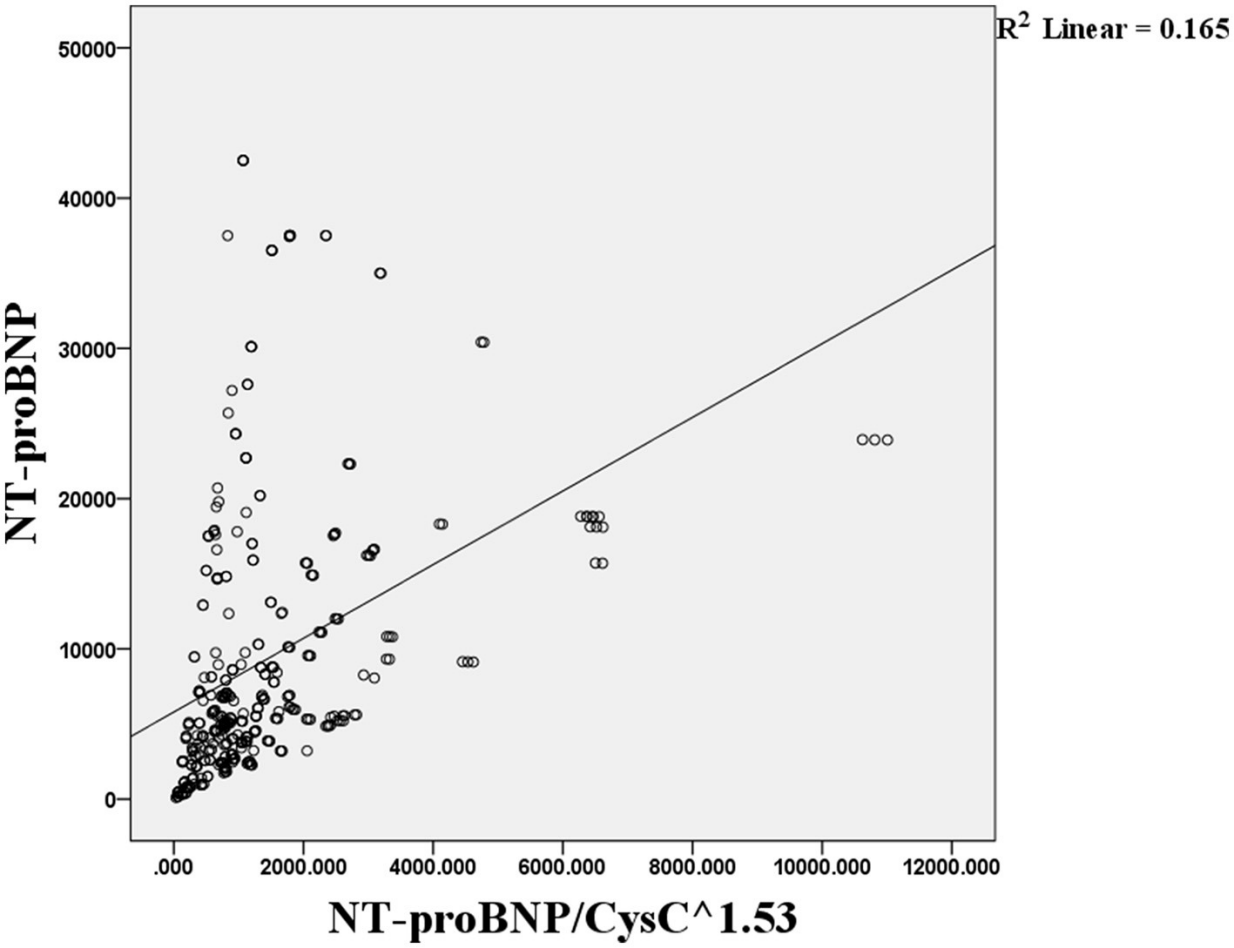
Pearson correlation analysis of the association between the NT-proBNP level and NT-proBNP/CysC^1.53^ ratio.

Pearson correlation analysis showed that the level of NT-proBNP was not correlated with the NT-proBNP/CysC^1.53^ ratio (*r* = 0.376, *p* = 6.909; Figure 1).

The E/e', GAS, GLS, LAVI, LVMI, serum urea, sCr, hs-cTnT, NT-proBNP, and NT-proBNP/CysC^1.53^ ratio in the HF group were significantly higher than those in the control group, while the LVEF was significantly lower than that in the control group (*p* < 0.05). No significant differences in age, CysC, LVDd, and LVDS were observed between the two groups (*p* > 0.05).

**Figure 2:**
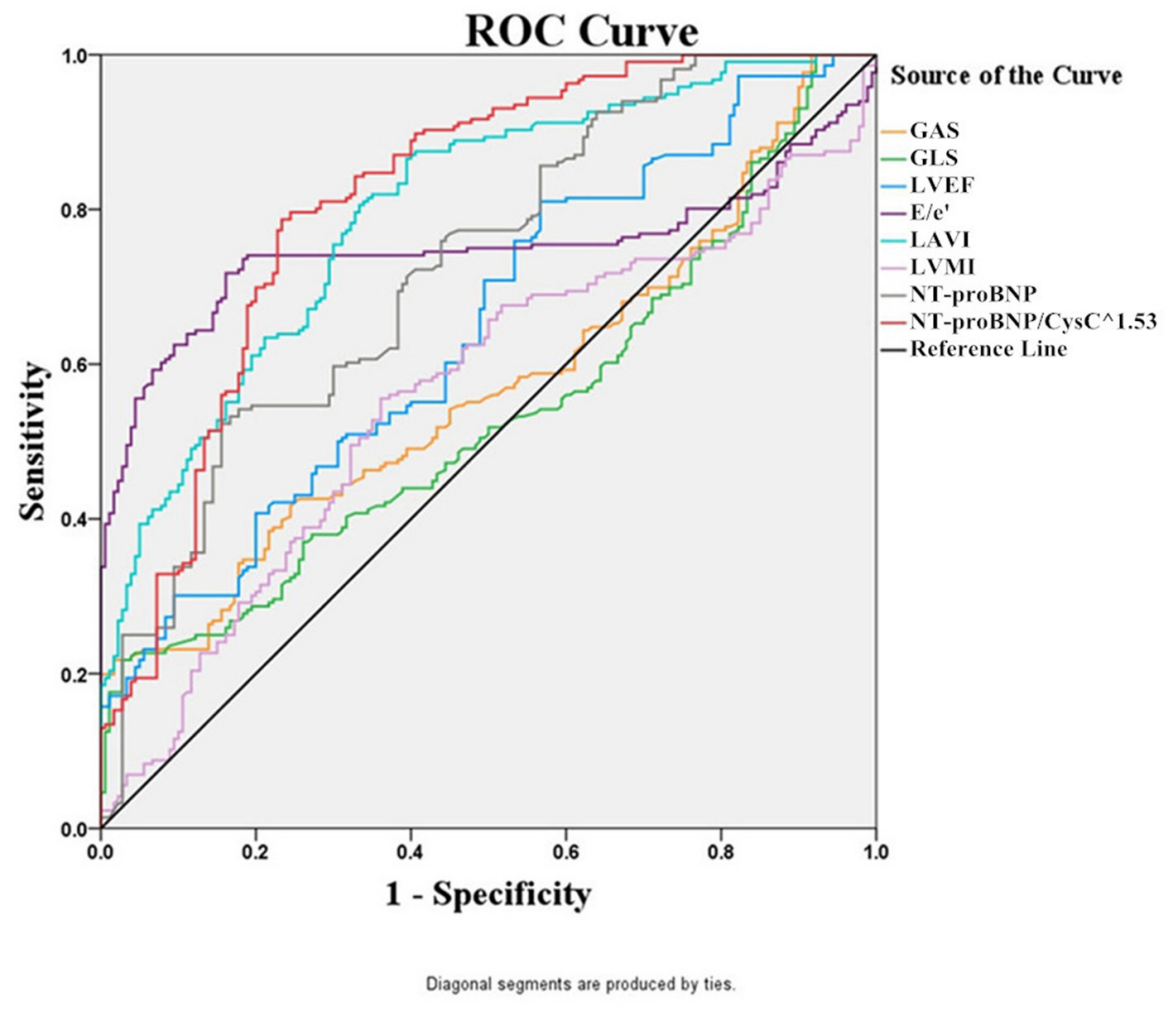
The ROC curves of NT-proBNP/CysC^1.53^, NT-proBNP, GAS, GLS, LVMI, LAVI, E/e', and LVEF for the diagnosis of HF in patients with CKD.

Then, we used ROC curve analysis to identify the predictors that were statistically different between the two groups. The ROC curves for eight potentially predictive factors (NT-proBNP/CysC^1.53^, NT-proBNP, GAS, GLS, LVMI, LAVI, E/e', and LVEF) were plotted. The following AUC values for the eight factors were calculated: AUC (NT-proBNP/CysC^1.53^) = 0.815, AUC (LAVI) = 0.798, AUC (E/e') = 0.747, AUC (NT-proBNP) = 0.726, AUC (LVEF) = 0.646, AUC (GAS) = 0.570, AUC (LVMI) = 0.561, and AUC (GLS) = 0.535. The AUC for the NT-proBNP/CysC^1.53^ ratio was greater than those for NT-proBNP and LVEF. The diagnostic cut-off value for the NT-proBNP/CysC^1.53^ ratio was 847.964, with a sensitivity of 78.24% and a specificity of 69.44%. The diagnostic cut-off value for NT-proBNP was 8,198 pg/mL, with a sensitivity of 52.31% and a specificity of 84.44% (Figure 2).

The clinical data of CKD patients from another local hospital were used to validate the new predictors and the truncation value. The baseline characteristics of 68 hospitalized patients with CKD are summarized in Table 2. The results showed a sensitivity of 70.27% and a specificity of 67.74%.

**Table 2:** Baseline clinical characteristics of CKD patients from another local hospital.

**Variables**	**HF group (*n* = 37)**	**Control group (*n* = 31)**	***t-value***	***p*-value**
Age (years)	69.459 ± 8.878	70.613 ± 10.990	0.479	0.223
Male [cases (%)]	23 (62.16)	18 (58.06)	0.118	0.462
BMI (kg/m^2^)	26.076 ± 2.838	25.419 ± 2.148	1.058	0.150
LVEF (%)	54.702 ± 12.168	60.111 ± 8.814	2.032	0.054
E/e'	19.232 ± 5.481	15.302 ± 1.995	3.501	0.000
GAS (%)	−24.687 ± 2.832	−26.786 ± 1.692	4.054	0.024
GLS (%)	−13.774 ± 2.011	−15.191 ± 2.261	3.598	0.033
LAVI (mL/m^2^)	40.070 ± 4.158	32.228 ± 4.032	3.357	0.071
LVMI (g/m)	113.238 ± 11.206	109.310 ± 10.619	1.474	0.377
Hb (g/L)	101.919 ± 25.537	112.871 ± 26.331	1.737	0.684
Urea (mmol/L)	16.132 ± 8.031	11.158 ± 4.599	2.542	0.012
sCr (μmol/L)	219.00 (127.00, 535.50)	232.50 (113.00, 301.50)	1.686	0.007
CysC (mg/L)	2.920 (1.820, 5.450)	3.010 (1.970, 3.620)	1.209	0.074
eGFR (mL/min/1.73 m^2^)	16.296 (7.802, 31.186)	17.076 (13.115, 31.560)	0.525	0.408
hs-cTnT (μg/L)	0.019 ± 0.007	0.016 ± 0.008	1.336	0.335
NT-proBNP (pg/mL)	9,301 (4,122, 13,005)	3,697 (936, 7059)	3.332	0.057
NT-proBNP/CysC^1.53^	1198.345 (736.270, 1937.597)	782.908 (242.424, 1255.494)	2.736	0.025

## Discussion

Renal function and cardiac function are interdependent in patients with CKD. HF has been identified as an independent risk factor for all-cause mortality in hospitalized CKD patients. The morbidity and mortality rates are high in CKD complicated with cardiac insufficiency and have been increasing in recent years (26). Thus, early diagnosis of cardiac insufficiency may improve the prognosis of patients with CKD.

HF can be divided into HFrEF, HFmrEF, and HFpEF. It has been reported that HFpEF accounts for more than half of all hospital admissions for HF (27). Early diagnosis of HFpEF is more difficult than that of HFrEF (LVEF ≤ 40%) and HFmrEF. The widely used New York Heart Association Classification System classifies HF based on subjective feelings of patients; therefore, the results may not be accurate. Even when the objective HFA-PEFF diagnostic algorithm is used, a cardiovascular ultrasound system (e.g., GE Vivid E90 Ultrasound System designed by GE Healthcare) is always needed for the classification of HF. However, in China, most primary and intermediate hospitals do not have such equipment. NT-proBNP is a diagnostic marker for HF, and its serum levels are closely associated with renal insufficiency and age. Therefore, the diagnostic cut-off value of NT-proBNP for HF should be adjusted based on the level of eGFR. However, no large-scale prospective clinical trials have provided an accurate cut-off value of NT-porBNP for the diagnosis of HF in patients with CKD (17).

By comparing the baseline characteristics of the HF and control groups, we found that the NT-proBNP/CysC^1.53^ ratio differed significantly between the two groups. Patients with HF showed a significantly higher NT-proBNP/CysC^1.53^ ratio than the control group, suggesting that a high NT-proBNP/CysC^1.53^ may be related to the occurrence of cardiac dysfunction in CKD. Our results also demonstrated that the NT-proBNP/CysC^1.53^ ratio was a more reliable predictor of HF than NT-proBNP, GAS, GLS, LVMI, LAVI, E/e', and LVEF in patients with CKD. According to the Kidney Disease: Improving Global Outcomes (KDIGO) 2012 clinical practice guideline for the evaluation and management of CKD (1), CysC is the best indicator of eGFR and has been used for eGFR calculation. Therefore, in this study, we used both NT-proBNP and CysC to evaluate the cardiac function of patients with CKD. To our knowledge, this is the first study to explore the potential of NT-proBNP combined with CysC as a predictor of HF in CKD patients.

A previous study (28) proposed that LVEF is an indicator of cardiac insufficiency in patients with early diagnosed CDK. However, the use of LVEF as a diagnostic marker cannot rule out cases with HFpEF. In the present study, patients with HFpEF, HFmrEF, and HFrEF were included in the HF group for the analysis of cardiac function in CKD patients, and thus, this study may provide more comprehensive results.

Gao et al. (29) proposed that the level of NT-proBNP can indicate HF in different eGFR intervals. However, whether the same cut-off value of NT-proBNP can be used for the diagnosis of HF over a wide range of eGFR values remains unknown. Moreover, they only included patients with HFrEF (LVEF ≤40%). In our study, both NT-proBNP and CysC levels were used to predict cardiac insufficiency, which may better reflect individual differences.

We further validated the results using the clinical data of patients from another local hospital. The NT-proBNP/CysC^1.53^ ratio accurately determined the cardiac function of these patients.

It should be noted that the diagnostic criteria that we have derived in this study were simpler than those recently published in ESC Chronic Heart Failure Guidelines 2021 and require fewer tests. In addition, laboratory test results have less interference and are more accurate than the results of ultrasound scans, and therefore, may have a wider range of applications.

The present study has some limitations. There were only 9 (2.27%) cases with stage 1–2 CKD. Due to compliance issues, patients with symptoms or signs of HF and with a HFA score between 2 and 4 did not undergo a diastolic pressure test or invasive hemodynamic measurement. Because there were only 12 (3.03%) cases with symptoms or signs of HF and with a HFA score between 2 and 4, it might not significantly affect the overall results. We only recruited a small number of Chinese subjects from another local hospital to validate the results, which may not represent the population in other areas. Taking into account the differences in regions, populations, and examinations (e.g., altitude, ethnicity, equipment, reagent), it is recommended that the current findings be validated in a local population. In summary, our study showed that the NT-proBNP/CysC^1.53^ ratio was a predictor of cardiac insufficiency in patients with CKD and might be used for the early detection of HF in this population in clinical settings.

## Data Availability Statement

The raw data supporting the conclusions of this article will be made available by the authors, without undue reservation.

## Ethics Statement

The studies involving human participants were reviewed and approved by Ethics Committee of Gezhouba Central Hospital of Sinopharm. The patients/participants provided their written informed consent to participate in this study.

## Author Contributions

SW, XW, and JL conceived and designed research. ML, CL, XY, HA, and TY collected data and conducted research. SW and YZ analyzed and interpreted data. YH and ML wrote the initial paper. SW, ML, YZ, and XL revised the paper. SW had primary responsibility for final content. All authors read and approved the final manuscript.

## Funding

This study was supported by the 2017 Three Gorges University Youth Science Foundation Project (No. KJ2017A011).

## Conflict of Interest

The authors declare that the research was conducted in the absence of any commercial or financial relationships that could be construed as a potential conflict of interest.

## Publisher's Note

All claims expressed in this article are solely those of the authors and do not necessarily represent those of their affiliated organizations, or those of the publisher, the editors and the reviewers. Any product that may be evaluated in this article, or claim that may be made by its manufacturer, is not guaranteed or endorsed by the publisher.

## Abbreviations

NT-proBNP: N-terminal pro B type natriuretic peptide
CKD: chronic kidney disease
CysC: cystatin C
ESC: European Society of Cardiology
ROC: receiver operating characteristic
eGFR: estimated glomerular filtration rate
HF: heart failure
BNP: Brain natriuretic peptide
ESRD: end-stage renal disease
AHF: acute heart failure
sCr: serum creatinine
hs-cTnT: highly sensitive cardiac troponin T
LVDd: left ventricular end-diastolic dimension
LVDs: left ventricular end-systolic dimension
LVEF: left ventricular ejection fraction
LMVI: left ventricular mass index
GAS: global area strain
GLS: global longitudinal strain
LAVI: left atrial volume index
FS: left ventricular fractional shortening
SV: stroke volume
E/e': the echocardiographic ratio of early diastolic mitral inflow velocity to early diastolic mitral annulus velocity
HFA: Heart Failure Association
PEFF: pre-test assessment; pre-test assessment; functional testing in case of uncertainty; final etiology
HFpEF: heart failure preserved ejection fraction
HFmrEF: heart failure with reduced ejection fraction
HFrEF: heart failure with reduced ejection fraction
AUC: area under the receiver operating characteristic curve
KDIGO: Kidney Disease: Improving Global Outcomes.
